# Phyllobilins: Emerging Bioactive Chlorophyll Metabolites and Their Potential Impact on Human Health

**DOI:** 10.3390/antiox15050629

**Published:** 2026-05-15

**Authors:** María del Rosario Serra, Antonio Pérez-Gálvez, María Roca

**Affiliations:** Group of Chemistry and Biochemistry of Pigments, Food Phytochemistry Department, Instituto de la Grasa (CSIC), 41013 Sevilla, Spain; mrserra@ig.csic.es (M.d.R.S.); aperez@ig.csic.es (A.P.-G.)

**Keywords:** anticancer, antioxidant, anti-inflammatory, bioactivity, chlorophyll, health, metabolism, phyllobilins, phytochemicals

## Abstract

Phyllobilins are chlorophyll metabolites that belong to bilin-type linear tetrapyrroles. Chlorophyll, the omnipresent green pigment from algae to higher plants, is essential for life on Earth, underscoring the significance of its metabolites among phytochemicals. Once largely overlooked, phyllobilins are now gaining recognition for their widespread presence in the human diet through the consumption of fruits and vegetables. This, together with their favorable bioavailability, has heightened the importance of elucidating their bioactive properties. Numerous studies have demonstrated their antioxidant and anticancer activities in vitro, as well as their ability to target actin. The anti-inflammatory effects of phyllobilins have also been demonstrated by evaluating their ability to inhibit the COX-2 pathway or attenuate the activation of the tryptophan–kynurenine pathway. The objective of this review is to highlight the value of phyllobilins by compiling current knowledge, with a particular emphasis on their bioactivity and potential impact on human health.

## 1. Introduction

In recent years, public interest in healthy eating has increased rapidly, and with it a widespread search for so-called functional foods and nutraceuticals. This trend is reflected in a growing body of literature on diet-related health [1], much of which focuses on phytochemicals and their potent bioactive properties. Given the vast diversity of phytochemicals, research has traditionally focused on carotenoids, polyphenols, isoprenoids, saponins, phytosterols, dietary fibers, and some polysaccharides [2]. Moving beyond these commonly studied phytochemical classes, recent research has begun to explore phyllobilins, a newly recognized group of plant metabolites that derive from chlorophyll and accumulate in cellular vacuoles [3].

Chlorophyll is essential for life on Earth, and the degradation of this green phytopigment gives rise to a spectacular and widely visible natural phenomenon. Remarkably, it is estimated that approximately 10^9^ tons of chlorophyll are degraded annually on land and in oceans [4]. The metabolites generated through this process—phyllobilins—therefore merit significant scientific attention as they do not undergo further metabolic transformation or degradation but instead accumulate in various configurations within plant cells. The consumption of fruits and vegetables that were green when immature thus suggests that phyllobilins are part of the human diet. Moreover, there is evidence that these chlorophyll metabolites can resist simulated gastrointestinal digestion and be assimilated by the intestinal Caco-2 cell model [5]. The consumption of fruits and vegetables that were green when immature thus suggests that phyllobilins are part of the human diet. Moreover, there is evidence that these chlorophyll metabolites can resist simulated gastrointestinal digestion and be assimilated by the intestinal Caco-2 cell model [5]. In this regard, once largely considered mere breakdown products, phyllobilins are now emerging as compounds of considerable relevance due to their remarkable structural diversity and recently uncovered health-promoting potential. Among their bioactive activities, they have been shown to exert antioxidant, anti-inflammatory, and anticancer effects.

Excessive production of reactive oxygen species (ROS) and reactive nitrogen species (RNS) in the human body can disrupt cellular redox balance. Both species lead to oxidative damage to major biomolecules such as proteins, DNA, and lipids [6]. This is of particular concern, as oxidative stress is responsible for the pathogenesis of numerous chronic diseases—including certain cancers—and is also closely associated with the aging process. In this context, antioxidants play a crucial role. For instance, in neurodegenerative diseases like Alzheimer’s, antioxidant compounds can help protect neurons from oxidative damage and slow disease progression [7]. Similarly, in cardiovascular conditions, antioxidants may help reduce oxidative stress and improve endothelial function, thus potentially preventing the risk of atherosclerosis and other heart-related issues [8]. Furthermore, antioxidants have demonstrated potential in mitigating diabetes-related complications by reducing oxidative stress and improving glycemic regulation [9]. Another important factor that contributes to the pathogenesis of many chronic diseases is chronic inflammation. Recently, considerable attention has been directed toward the development of phytochemical-based anti-inflammatory drugs to reduce the side effects associated with conventional therapies [10].

Within this framework, phyllobilins represent a highly promising focus for advancing both healthy nutrition strategies and the development of novel pharmaceuticals. Accordingly, the objective of this review is to consolidate current knowledge on this emerging group of phytochemicals, with special focus on its bioactive properties, thereby underscoring their importance and potential.

## 2. Chemistry of Phyllobilins: Structures and Metabolism

**Figure 1:**
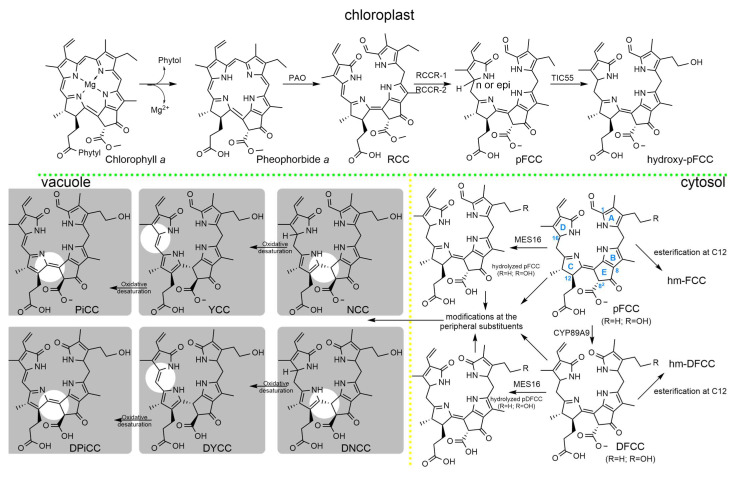
Chlorophyll metabolism in plants.

Leaf senescence and fruit ripening share a largely common pathway that transforms chlorophyll molecules into a structurally diverse family of linear tetrapyrrolic catabolites known as phyllobilins. These compounds can be grouped into several sub-families according to the structural features that arise along the pathway. In the chloroplastic phase of chlorophyll breakdown, pheophorbide *a* is the immediate substrate for a two-step enzymatic process that yields a primary blue-fluorescing chlorophyll catabolite (pFCC) that shows the same tetrapyrrolic skeleton of the parent molecule, specifically the isocyclic ring with its characteristic keto and carboxymethyl groups, as well as the rest of the peripheral substituents observed in pheophorbide *a* (Figure 1).

The initial crucial structural change is the oxygenolitic opening of the macrocycle at the α-methine bridge, generating a linear tetrapyrrole with the introduction of formyl and keto groups at C1 and C19 positions, respectively. In that way, the intermediate generated contains two oxygen atoms and four hydrogen atoms more than the parent pheophorbide *a*. This step is performed by the pheophorbide *a* oxygenase (PaO) enzyme, yielding the red chlorophyll catabolite (RCC). RCC is then reduced at the C15-C16 double bond by the activity of the RCC reductase enzyme. Plants contain one of the two stereospecific RCCR lineages, RCCR-1 and RCCR-2. This step introduces a configurational difference at C16, leading either to the normal series (*n*-pFCC) or to the epimeric series (*epi*-pFCC) (Figure 1). The reached configuration is retained during the complete transformation route to the final catabolites.

The starting intermediate, pFCC, is the basic element for additional enzymatic/chemical reactions that array new structures to the peripheral groups and build up the phyllobilins family. Every year, new members are added to this family.

A first remarkable change in the canonical pFCC structure still may take place within the chloroplast via an enzymatic reaction catalyzed by the TIC55 enzyme that hydroxylates the ethyl side chain at the C3^2^ [11]. This enzymatic process is frequent [12], although not essential in the chlorophyll catabolic pathway, and allows for further transformations of the already modified pFCC at the C3^2^. Indeed, this change potentially influences the reactivity of other peripheral positions by promoting or hindering changes subsequently in the pathway, as denoted below. From this point, the additional changes either in the pFCC or in its C3^2^-hydroxylated derivative occur after export from the chloroplast, mainly in the cytosol compartment and later in the vacuole, where the ‘final’ phyllobilins are accumulated (Figure 1).

Two of the modifications that stand out are the oxidative deformylation of the formyl group at C1 and the demethylation of the carboxymethyl group at the isocyclic ring (Figure 1). These modifications highlight, among others, that they are enzymatic, and the enzymes involved have been characterized. The former is catalyzed by cytochrome P450 monooxygenase, CYP89A9, which is responsible for the accumulation of dioxobilin-type phyllobilins [13]. It is with the production of this dioxobilane-type phyllobilin that the two-family branches separate: pFCCs that elude the activity of CYP89A9, type-I phyllobilins (1-formyl-19-oxophyllobilanes), and pFCCs that follow this oxidative deformylation, type-II phyllobilins (1,19-dioxophyllobilanes). The expression of this enzyme is species-specific, and the generation of dioxobilane-type phyllobilins is compatible with both pFCC substrates exported from the chloroplast. However, it is suggested that enzymatic deformylation is more selective towards C3^2^-hydroxylated pFCC and its conversion is even faster [14]. This enzymatic transformation seems to take place by the insertion of one oxygen atom between the C1 and the attached formyl group. The subsequent hydrolysis of the formate ester and protonation at C4, changes the ring A from the original pyrrole arrangement to a pyrroline one, the same configuration as that of ring D in the pFCCs generated in the chloroplast.

The second key cytosolic modification is the enzymatic demethylation of the carboxymethyl group at C8^2^, which is performed by the cytosolic enzyme MES16 [15]. MES16 activity is dependent on prior C3^2^ hydroxylation by TIC55 and therefore primarily acts on FCCs that have already been modified in the chloroplast [11]. Indeed, the activity of this enzyme might kinetically compete with CYP89A9 deformylation. If the pFCC exported from the chloroplast, either hydroxylated or not at the C3^2^, is eventually demethylated before oxidative deformylation, then accumulation of type-I phyllobilins seems to be the preferred route [13]. Demethylation of the carboxymethyl group is the primary modification of the chlorophyll breakdown route taking place at the characteristic isocyclic ring, which is a highly reactive site for the parent pigments of this process, chlorophyll/pheophorbide, particularly in reactions involving ROS.

Structural modifications might then occur (Figure 1), increasing the structural diversity of both type-I and type-II phyllobilins. These include dihydroxylation of the vinyl group at the C18 position, the single hydroxylation of the methyl group at C2, which has been exclusively detected in senescent leaves of *Arabidopsis thaliana*, so far [16]. Solely type-II phyllobilins can be modified with the introduction of a hydroxymethyl group at C2 or at C4 [17]. In correlation with the preference of CYP49A9 towards C3^2^-hydroxylated pFCC, the hydroxymethylation at the C2/C4 position seems to find a purpose for the C1 fragment via folate-based enzyme [14].

The introduction of hydroxyl groups, particularly with the initial TIC55-hydroxylation in the chloroplast at C3^2^, but also at C18 in ring D, and the original existence of a free propionic group at C12, might induce esterification at those positions. Phyllobilins with the C3^2^ hydroxyl group esterified are frequently described as products of the chlorophyll catabolism. Diverse structures might participate in this modification through glycosylation, which has also been described in the hydroxyl at C18^2^ [18], malonylation, and combinations of both (Figure 1). Even singular structures arising from the esterification with the same structure (glucopyranosyl group) linking the hydroxyethyl at C3 and the propionic acid at C12, generating highly specialized conjugates, have been described [19].

A different mark is achieved if the esterification involves the propionic acid group at C12 in ring C. The free propionic group is mechanistically involved in the non-enzymatic isomerization of the modified FCCs/DFCCs related so far to the corresponding isomers, NCCs/DNCCs, which finally accumulate in the vacuole. This acid-induced isomerization produces an intramolecular protonation at C10, preferentially with an R configuration. At the same time, it causes the reorganization of the π system in ring C, which changes from a pyrroline-type to a pyrrole-type arrangement. The free propionic group starts and drives this isomerization process. In this sense, the esterification at this position greatly slows the conversion rate and reduces stereoselectivity at C10, compromising the final formation of NCCs/DNCCs.

The fate of the modified FCCs with a free propionic acid chain at C12 is the acid-induced isomerization to yield NCCs/DNCCs accumulated in the vacuole. The modified FCCs fraction that switches to esterification at C12^3^ escapes rapid isomerization. They may accumulate as persistent fluorescent catabolites, the so-called hypermodified FCCs (hmFCCs), as it has been described in the peels of ripe bananas [20]. Indeed, hmFCCs are characterized by esterification at C12^3^ with unusual esterifying structures, such as daucyl group [21] or glucopyranosyl–hydroxytyrosyl moieties [22,23]. Nevertheless, traces of NCCs esterified at the C12^3^ have been described in peels of ripe bananas [21].

These structural differences are directly reflected in the spectroscopic properties of the phyllobilins sub-families related so far. The UV–visible spectrum of the FCCs displays a characteristic absorption maximum around 360 nm, arising from the chromophore extension across rings B and C. Such a maximum is associated with the featuring absorption maximum of ring A observed at 315 nm, consistent with the 1-formyl-19-oxo arrangement of ring A. Additionally, FCCs display a blue fluorescence with emission maximum at 450 nm, which also features the hypermodified FCCs. In the case of NCCs, the single maximum at 315 nm is the original mark detailing the 1,19-formyl arrangement [3]. This maximum is obviously absent in the case of DNCCs that do not show a particular characteristic UV–visible spectrum, which makes them less readily recognized by UV–visible spectroscopy alone.

The transformations of the chlorophyll breakdown route do not stop with the formation of NCCs and DNCCs. Still, two processes are possible since these colorless phyllobilins may undergo further oxidative transformations that extend the conjugated system and generate colored derivatives. The introduction of an unsaturation at the C15-C16 bond yields the yellow chlorophyll catabolites (YCCs/DYCCs). This process implies a reversion of the RCC reductase function at the initial steps of the route and the extension of the chromophore between rings C and D (Figure 1). The products of this process contribute to the characteristic coloration of senescent vegetal tissues in autumn. Consequently, the UV–visible spectrum of yellow chlorophyll catabolites is characterized by a maximum at 430 nm [24]. The second oxidation process, following the transformation to YCCs/DYCCs, is the desaturation at the C10-C11 bond with redistribution of the double-bonds in ring C (Figure 1). This oxidative desaturation yields the pink phyllobilins (PiCCs), which means further extension of the chromophore and produces an absorption band near 520 nm [24].

The complete route described above and depicted in Figure 1 is commonly known as the PaO/phyllobilin pathway, and many of the modifications occurring within it affect the polarity of the resulting chlorophyll metabolites. Among these modifications, hydroxylation at the C3^2^ position or hydroxymethylation at C2/C4 increases the polarity of phyllobilins, as does the deformylation of the formyl group at C1. In contrast, polarity decreases upon oxidation of (D)NCCs to (D)YCCs. Polarity is an important factor to consider, as it may influence the bioavailability and bioactive properties of phyllobilins.

## 3. Occurrence and Distribution

To date, around 200 phyllobilins have been described, but this figure includes repeated compounds identified in different species. This confusion stems from the initial nomenclature system, in which the same compound received different names depending on the species in which it was identified. In addition, a few of these phyllobilins are chemical “artifacts,” several have been identified in mutant organisms (e.g., *Arabidopsis thaliana mes16* mutant [15,17]), and an important figure has been in vitro oxidized [25,26,27], esterified at the C12^3^ position [21,28,29], or decarboxylated at C8^2^, yielding pyro-derivatives [30]. Additionally, in vitro metal complexes of phyllobilins have been formed [31,32,33]. Therefore, considering only phyllobilins naturally formed during the senescence or ripening in photosynthetic organisms, the number drops to 66 different phyllobilins.

Table 1 lists all these phyllobilins, which are plausible compounds to be found in foods; in fact, several of them have been identified in fruits and vegetables. The general name consists of the phyllobilin type (FCC, DFCC, etc.), preceded by “hm-”, “pyro”, or “bc-” when the compound is hypermodified, pyro or bicyclic, respectively, and followed by its isotopic mass [34]. For clarification, besides the general name for each compound, Table 1 also includes the alternative names that the same phyllobilin could have been received along the bibliography.

As phyllobilins are biosynthesized from chlorophylls, all photosynthetic organisms are theoretically candidates for synthesizing phyllobilins. However, experimentally, phyllobilins have not been identified in every chlorophyll-containing species; otherwise, phyllobilin presence seems to be limited to specific phylogenetic groups. In fact, it can be considered that, at least for the moment, the identification of these 66 phyllobilins is almost restricted to angiosperms [35], formerly classified as the division Magnoliophyta, but modern phylogenetics treats them as a major clade known as the Angiospermae. Angiosperms are the largest, most diverse group of plants, characterized by bearing flowers and enclosing seeds within a fruit. However, apart from this clade, the identification in other phylogenetic groups is null, except for a representative in one species of the phylum Chlorophyta [36] and in one species of the old phylum Pteridophyta (actual clade Monilophyta), specifically in a fern [27]. Specifically, although chlorophyll degradation is induced in the green alga *Auxenochlorella protothecoides* (formerly *Chlorella protothecoides*) under nitrogen-limited heterotrophic dark conditions, it exhibits, in contrast to land plants, a truncated pathway that culminates in red chlorophyll catabolites, without further progression along the pathway. Whereas the upstream segment of the pathway is strongly conserved [37], downstream phyllobilin modifications do not appear to be required for plant viability. It remains challenging to attribute a specific physiological function to each phyllobilin molecule [38].

Overall, the accumulation patterns of phyllobilins across different divisions, classes, and families merit further investigation. Although the fate of chlorophyll in species that do not accumulate detectable levels of phyllobilins remains unresolved, elucidating the distribution of specific phyllobilins across defined phylogenetic lineages is expected to provide valuable molecular insights into the regulation and diversification of the chlorophyll degradation pathway. Such an approach will facilitate a deeper understanding of the factors underlying the distribution patterns of phyllobilins, helping to explain why, as shown in Table 1, some are restricted to a single species, whereas others display a remarkably broad occurrence, having been identified in up to 18 species.

Concomitantly, the majority of phyllobilins have been reported in senescent leaves, likely not due to a higher intrinsic distribution, but rather because early research efforts predominantly focused on this part of the plant. More recently, however, phyllobilins have also been identified in fruits and vegetables. While there has been ongoing debate regarding the similarity of chlorophyll degradation pathways across different organs, current evidence suggests that fruits and vegetables exhibit a greater structural diversity of phyllobilins compared to senescent leaves [34].

Among the structurally characterized phyllobilins (Table 1), DNCCs and NCCs constitute at least 50% of the metabolites formed and accumulated in vivo. These are followed in abundance by yellow and fluorescent chlorophyll catabolites. In contrast, other subclasses—such as PiCCs, DYCCs, DFCCs, and iPBs—are only sporadically reported in natural systems. This uneven distribution is primarily governed by metabolic feasibility, reflecting the thermodynamic and kinetic preference for specific biochemical transformations under physiological conditions. Notwithstanding, the identification of novel phyllobilin structures is advancing in parallel with the development of increasingly sensitive and selective analytical methodologies. Accordingly, the number of phyllobilins reported in Table 1 is expected to expand in the coming years, particularly in light of recent progress in analytical platforms and database resources.

## 4. Bioactive Properties

### 4.1. Antioxidant

**Figure 2:**
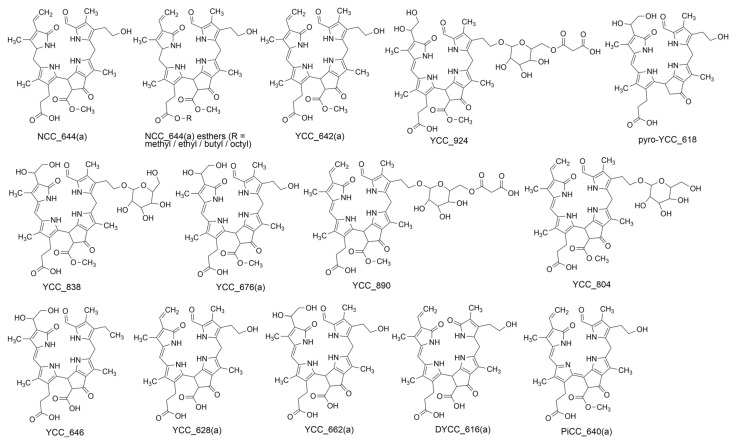
Structures of phyllobilins studied for their bioactivity and health effects. Phyllobilins with antioxidant activity: all structures depicted in this figure [39,40,41,42,44,46]. Phyllobilins with anti-inflammatory activity: YCC_662(a), YCC_628(a), YCC_642(a), and YCC_804 [40,42,44]. Phyllobilins with anticancer activity: YCC_642(a), esters of NCC_644(a), and PiCC_640(a) [28,52]. Actin-targeting phyllobilins: YCC_642(a), and PiCC_640(a) [52].

The study of the antioxidant properties of phyllobilins has only recently emerged as a research focus; except for a single earlier report, most investigations in this field have been conducted within the past five years (Table 2). To date, these studies have predominantly relied on in vitro antioxidant assays (e.g., ORAC, FRAP, and DPPH) or on the evaluation of intracellular ROS scavenging capacity, typically assessed using the DCFH-DA fluorescent probe. Most investigations have focused on the YCC configuration; however, other structural motifs have also been examined. In this sense, Müller et al. (2007) were the first and the only to test the antioxidant activity of an NCC phyllobilin [39], *Pc*-NCC-1 (i.e., NCC_644(a), according to the new nomenclature) (Figure 2), which was isolated from freshly cut yellow peels of mature “Williams” pears (*Pyrus communis*) (Table 2). This NCC exhibited an antioxidant activity very close to that of bilirubin, a remarkable antioxidant that is the catabolite of the tetrapyrrolic heme and is therefore structurally very similar to phyllobilins. This same natural compound, NCC_644(a), was later isolated from senescent leaves of *Cercidiphyllum japonicum*, and its antioxidant capacity was again tested using the ORAC assay [40] (Table 2). The obtained ORAC value was 2.23 ± 0.13 Trolox equivalents, thus demonstrating a high peroxyl-radical scavenging capacity and supporting the results of the previous 2007 study.

Nevertheless, as noted above, yellow chlorophyll catabolites constitute the most extensively studied class of phyllobilins. For example, Karg et al. investigated YCC_642(a) (Figure 2) in senescent leaves of *Cercidiphyllum japonicum* [40], the oxidation product of NCC_644(a), which proved to be the most active compound (4.67 ± 0.32 Trolox equivalents). This study further analyzed the antioxidant potential of these two phyllobilins in living cells by measuring intracellular ROS formation in Caco-2 cells using the 2′,7′-dichlorofluorescein diacetate (DCFH-DA) probe. Cells pre-treated with NCC_644(a) or YCC_642(a) showed a dose-dependent reduction in ROS activity. YCC_642(a) exhibited the strongest effect: it could reduce ROS formation by almost 80% at the highest concentration tested. In parallel, six different YCCs (Table 2) from the medicinal plant *Echinacea purpurea*, and their antioxidant potential, were evaluated using the FRAP assay [41]. Five out of the six YCCs exhibited significantly stronger antioxidant activity than caffeic acid—a well-known antioxidant that is present in *Echinacea* extracts—and, interestingly, their antioxidant potency decreased in parallel with the polarity of the YCCs, with YCC_924 (Figure 2) showing the highest activity, more than three times that of caffeic acid. These compounds were also shown to be taken up by human endothelial kidney (HEK) cells, and were therefore shown to be stable once taken up.

Considering the stability of YCCs in human cells, their *in-cellulo* anti-oxidative activity was also tested. Remarkably, the three tested YCCs—YCC_924, YCC_676(a), and YCC_804 (Figure 2)—were able to scavenge ROS in stimulated HeLa cells and protect them from oxidative stress, as assessed by total cellular GSH levels, both assays conducted at concentrations as low as 10 µM. In a subsequent study, Karg et al. (2021) isolated the yellow chlorophyll catabolite *Ud*-YCC [42], designated YCC_804 (Figure 2), from fresh senescent nettle leaves—one of the same phyllobilins previously analyzed in Echinacea purpurea (Table 2). They characterized its physiological activities alongside major bioactive phytochemicals reported in nettle leaves, including caffeic acid, chlorogenic acid, rutin, and quercetin. In an in vitro FRAP assay, YCC_804 exhibited antioxidant activity comparable to that of caffeic acid, chlorogenic acid, and rutin, and significantly higher than Trolox. Furthermore, YCC_804 was again shown to efficiently scavenge intracellular ROS, this time in stimulated HEK cells, in a similar range as even higher concentrations of caffeic acid, chlorogenic acid, and rutin. These results corroborate and extend the findings reported in Karg et al. (2019) [41]. Interestingly, the same YCC was also identified in freshly brewed tea prepared from various commercial nettle tea bags, suggesting that this chlorophyll catabolite may contribute to the well-known antioxidant effects of nettle tea [43].

Continuing the investigation of the potential antioxidant activity of yellow chlorophyll catabolites, four YCCs obtained from senescent leaves of the medicinal plant garden nasturtium were evaluated using the FRAP assay [44] (Table 2). YCC_646 (Figure 2) exhibited an antioxidant capacity three times stronger than the vitamin E derivative Trolox, while YCC_662(a) activity was two times stronger than Trolox. Moreover, both compounds demonstrated antioxidant activity comparable to that of chlorogenic acid and isoquercitrin, two well-known polyphenolic compounds present in nasturtium leaves. The remaining two YCCs—pyro-YCC_618 and YCC_628(a) (Figure 2)—exhibited antioxidative capacities similar to that of Trolox. Additionally, an *in cellulo* ROS assay performed with YCC_662(a) and YCC_628(a) yielded promising results at low micromolar concentrations: a reduction of 36% and 39% were observed, respectively, in ROS production relative to the positive control (H_2_O_2_-treated cells). These activities are comparable to those of chlorogenic acid, suggesting that YCCs from nasturtium leaves may be involved in the health benefits associated with nasturtium as a medicinal plant. And more recently, Elvert et al. (2025) fractionated extracts from senescent leaves of *Salvia officinalis* and evaluated their antioxidant potential using a FRAP assay and an intracellular ROS-scavenging assay [45]. Each fraction contained phyllobilins, although these were not present in purified form. Based on the results, the 13 fractions obtained were classified as “inactive”, “active”, or “very active” using a color legend. Overall, the findings indicate the presence of potentially bioactive phyllobilins in *Salvia officinalis* leaf extracts, with *Sao*-YCC (i.e., YCC_728) detected in fractions classified as “active” or “very active” in the FRAP assay.

Notably, PiCCs have been analyzed only in the last-mentioned study [45], and were predominantly found in “inactive” fractions. This limited redox activity of PiCCs may be attributed to their largely unsaturated structure. Nevertheless, direct structure–activity relationship conclusions remain difficult, as different outcomes were obtained depending on the assay used (FRAP versus cellular ROS assay).

Finally, despite the notable structural features of dioxobilin-type chlorophyll catabolites, these compounds have not been extensively studied. To date, research has been largely confined to DYCC. In this context, DYCC_616(a) (commonly known as *Bos*-DYCC) (Figure 2, Table S1), isolated from senescent leaves of savoy cabbage, is the phyllobilin that most closely resembles bilirubin, exhibiting an identical “western hemisphere” [46]. Notably, this DYCC showed significantly higher antioxidative potential compared to bilirubin in two different in vitro assays: FRAP and DPPH [46] (Table 2).

Collectively, these findings suggest that phyllobilins, alongside other phytochemicals, may contribute to the well-established antioxidant benefits associated with the consumption of fruits and vegetables [47,48], as well as the use of medicinal plants [49], highlighting their potential relevance in preventing and managing oxidative stress-related disorders.

**Table 2:** Summary of antioxidant assays performed to date on phyllobilins. Structures of the studied phyllobilins are depicted in Figure 2.

Antioxidant Capacity			
Reference	Plant Source	Tested Phyllobilins	Assay
[39]	*Pyrus communis*	NCC_644(a)	In vitro antioxidant activity (AIBN-induced linoleic acid peroxidation assay)
[40]	*Cercidiphyllum japonicum*	NCC_644(a), YCC_642(a)	In vitro antioxidant activity (ORAC assay)
			Intracellular ROS scavenging ability (Detection of intracellular ROS with DCFH-DA dye)
[41]	*Echinacea purpurea*	YCC_924, YCC_838, YCC_676(a), YCC_890, YCC_804, YCC_642(a)	In vitro antioxidant activity (FRAP assay)
		YCC_924, YCC_676(a), YCC_804	Intracellular ROS scavenging ability (Detection of intracellular ROS with DCFH-DA dye)
			Intracellular protective effects from oxidative stress (Detection of intracellular GSH with NEM)
[42]	*Urtica dioica*	YCC_804	In vitro antioxidant activity (FRAP assay)
			Intracellular ROS scavenging ability (Detection of intracellular ROS with DCFH-DA dye)
[44]	*Tropaeolum majus*	YCC_662(a), pyro-YCC_618, YCC_646, YCC_628(a)	In vitro antioxidant activity (FRAP assay)
		YCC_662(a), YCC_628(a)	Intracellular ROS scavenging ability (Detection of intracellular ROS with DCFH-DA dye)
[47]	*Salvia officinalis*	Non-purified fractions containing phyllobilins	In vitro antioxidant activity (FRAP assay) Intracellular ROS scavenging ability (Detection of intracellular ROS with DCFH-DA dye)
[46]	*Brassica oleracea* var. *Sabauda*	DYCC_616(a)	In vitro antioxidant activity (FRAP and DPPH assays)

### 4.2. Anti-Inflammatory

The principal mechanisms through which phytochemicals exert anti-inflammatory effects include the inhibition of pro-inflammatory cytokines (e.g., TNF-α, IL-6, and IL-1β), suppression of inducible nitric oxide synthase (iNOS) and the consequent reduction in nitric oxide (NO) production, as well as the modulation of key inflammatory signaling pathways such as NF-κB (nuclear factor kappa B) and MAPK. However, at present, there is no experimental evidence describing the in vivo mechanisms of action of phyllobilins. Nevertheless, initial studies have begun to address their potential anti-inflammatory activity, at present restricted to the YCC configuration. Several, established approaches have been used, such as evaluating the inhibition of the COX-2 pathway and emerging targets, such as the tryptophan–kynurenine pathway (Table 3).

Using the fluorometric in vitro cyclooxygenase inhibition assay, Karg et al. (2021) investigated the anti-inflammatory effect of YCC_804 (Figure 2) isolated from freshly senescent nettle leaves [42] (Table 3). The capacity of YCC_804 to inhibit the pro-inflammatory enzyme COX-2 was comparable to that observed for the positive controls, caffeic acid and chlorogenic acid—two of the main phytochemicals found in nettle. Regarding COX-1, it was also inhibited by the yellow chlorophyll catabolite at micromolar concentrations, although to a lesser extent than by caffeic and chlorogenic acids. The effect of YCC_804 on COX-2 protein expression levels was further assessed in the macrophage cell line J774A.1. At a concentration of 50 µM, YCC_804 significantly reduced COX-2 expression levels—an effect not observed for caffeic acid. Moreover, an ELISA assay measuring prostaglandin E_2_ (PGE_2_) production confirmed the anti-inflammatory activity of YCC_804, as well as of the other tested nettle phytochemicals. Nettle leaf extracts have previously been shown to reduce inflammatory responses through various mechanisms, including the inhibition of the biosynthesis of arachidonic acid cascade enzymes, especially COX-1 and COX-2 [43]. In fact, nettle tea or herbal supplements have been shown to effectively minimize the symptoms of inflammatory diseases such as rheumatoid arthritis and chronic myalgia. Karg et al. (2021) also demonstrated that the phyllobilin YCC_804 was stable during a simulated gastric and intestinal digestion, both in a pure form as well as in nettle tea [42]. Taken together, these findings suggest that the chlorophyll catabolite YCC_804 may contribute to the anti-inflammatory effects of nettle [43].

Using the same experimental approach, Frei et al. (2024) evaluated YCC_662(a) and YCC_628(a) (Figure 2), isolated from the medicinal plant garden nasturtium, for their anti-inflammatory activity [44] (Table 3). At low micromolar concentrations, both compounds exhibited inhibitory effects on COX-1 and COX-2 activity. Comparison of COX-1 dose–response curves revealed that YCC_628(a) exhibited a pronounced inhibition, with an IC_50_ of 8 µM. Regarding COX-2 inhibition, both YCCs showed a presumably stronger inhibitory potential than chlorogenic acid, a well-known polyphenolic compound of nasturtium leaves. On the protein level, in LPS-stimulated macrophages, YCC_628(a) significantly reduced COX-2 expression, whereas YCC_662(a), chlorogenic acid, and isoquercitrin showed no significant effect. Collectively, the discovery of these two yellow chlorophyll catabolites in nasturtium leaves as potent cyclooxygenase inhibitors expands the bioactive phytochemical repertoire of this medicinal plant.

When immune cells such as human peripheral mononuclear cells (PBMCs) are stimulated, they activate the enzyme indoleamine 2,3-dioxygenase-1 (IDO-1), which is involved in catalyzing the conversion of tryptophan into kynurenine [50]. The tryptophan–kynurenine pathway is therefore activated in response to inflammatory stimuli and represents one of the most important immunometabolic pathways of the cellular immune response. Indeed, the activity of this pathway serves as a key biomarker in several human disorders associated with immune activation, such as infections [51], and is considered to be an emerging and highly relevant method for assessing the anti-inflammatory effects of phytochemicals and natural compounds. This approach has been applied to determine the influence of the yellow chlorophyll catabolite YCC_642(a) (Figure 2) isolated from senescent leaves of *Cercidiphyllum japonicum* on the inflammation-induced tryptophan metabolism in PBMCs [40] (Table 3). Upon addition of YCC_642(a) to PHA-stimulated PBMC, tryptophan concentrations in the supernatants increased in a dose-dependent manner, whereas kynurenine levels decreased. This indicates that YCC_642(a) may attenuate the activation of IDO-1 and, consequently, the tryptophan–kynurenine pathway, thereby suggesting an anti-inflammatory potential. Further studies are warranted to elucidate the underlying molecular mechanisms of this effect.

Taken together, the evidence indicates that the four tested YCCs—YCC_804, YCC_642(a), YCC_662(a), and YCC_628(a)—exhibit consistent anti-inflammatory effects, suggesting that this specific subgroup represents a particularly promising class of anti-inflammatory phytochemicals.

### 4.3. Anticancer

Building on the previously described bioactive properties of phyllobilins, the next step was to evaluate the potential anticancer effects of these phytochemicals. This research area is still in its early stages, but it represents a promising field of investigation in which significant advances are expected. Although only a limited number of studies have been published to date, the available results are highly informative, not only identifying phyllobilin configurations with potential anticancer activity, but also indicating the stages of cancer progression at which phyllobilins may exert a biological effect.

Concerning cytotoxicity, it seems that the NCC configuration does not show any significant inhibition of cell proliferation, at least for the two epimers of NCC_644(a), isolated from senescent leaves of the katsura tree and the plane tree [28]. On the contrary, the YCC configuration exhibited cytotoxic effects against cancer cells, as demonstrated for YCC_642(a) (Figure 2), the oxidation product of NCC_644(a), and a less polar phyllobilin derivative (Table 4).

The antiproliferative activity of phyllobilins has been conducted on two different human cancer cell lines, the urinary bladder carcinoma T24 cell line and the highly invasive epithelial breast cancer MDA-MB-231 cell line, testing both NCC and YCC configurations. In a subsequent experiment, the compound examined was the oxidation product of NCC_644(a), specifically YCC_642(a). In contrast to NCC, YCC (YCC_642(a)) exhibited a pronounced anti-proliferative effect in both cell lines, with IC_50_ values of 4.6 µM against T24 cells and 7.0 µM against MDA-MB-231 cells. Given these promising results, the effect was further examined using a colony formation assay, in which YCC_642(a) significantly inhibited the long-term survival and proliferation of cancer cells. Moreover, annexin V/PI staining assay demonstrated that this chlorophyll catabolite induced cell death in a dose-dependent manner and acted as a potent inducer of apoptotic cell death. Additional cell cycle analysis of T24 and MDA-MB-231 cells was conducted after different treatments with YCC_642(a), revealing a marked arrest of cells in the G2/M cell cycle phase and a significant increase in cells in the sub-G_1_ phase. The latter confirms that YCC_642(a) promotes apoptotic cell death, corroborating the results of the annexin V/PI assay. Taken together, these findings identify YCC_642(a) as a potent anticancer agent in human cells.

Recent efforts have also focused on elucidating and enhancing the antiproliferative activity of phyllobilins (Table 4). For such an aim, NCC (NCC_644(a)) has been chemically modified by esterifying the propionic acid side chain at C12 with alkyl groups of increasing length [28], decreasing their polarity (Figure 2). Notably, esterification of that position also occurs naturally, for instance, in banana peels [21]. Remarkably, this structural modification influenced the inhibitory effects on the proliferation of T24 and MDA-MB-231 cells. The antiproliferative activity increased with the chain length of the alkyl esters, with the octyl ester displaying the lowest IC_50_ values: 3.4 µM against T24 cells and 16.3 µM against MDA-MB-231 cells. This suggests that phyllobilins with lower polarity exhibit higher antiproliferative activity. This enhanced efficacy may be primarily due to increased cellular membrane permeability, as lipophilic compounds cross membranes more easily.

Therefore, the polarity of phyllobilins may play a key role in their cytotoxic potential, and, importantly, they open new perspectives for the rational modification of chlorophyll catabolites to enhance their bioactive and potential pharmacological properties. It is known that all tested phyllobilins can be taken up by cancer cells and remain stable under the established experimental conditions [28]. More interestingly, when treating cells with NCC-esters, they were detected at higher concentrations, and only minute amounts of hydrolysed “free” NCC_644(a) were observed. This finding indicates that the observed antiproliferative effects of the NCC-esters do not rely on the release of the “free” NCC_644(a), but rather that the esterified forms exhibit this activity themselves.

In the context of cancer, cell migration is one of the initial steps leading to tumor invasion and metastasis. Consequently, the identification of phytochemicals capable of reducing cellular motility represents a promising strategy for anticancer intervention. Using a scratch wound-healing assay in two human cancer cell lines (T24 and HeLa cells), the influence of four linear tetrapyrrolic compounds on cell migration (YCC, PiCC, bilirubin, and biliverdin) has been examined [52] (Table 4). Interestingly, all of them inhibited cell migration to a different extent without altering cell viability, with PiCC_640(a) exhibiting the potent effect even at low nanomolar doses. Therefore, compounds capable of reducing cellular motility—YCC_642(a) and PiCC_640(a), in this case—may be regarded as potential antimetastatic agents [53].

### 4.4. Other Bioactive Properties and Functions

As described above, one of the potentialities of tetrapyrroles is their effect on cell mobility [52], and, considering that the cytoskeletal protein actin plays a central role in cell migration [54], the effects of four anti-migratory compounds—YCC, PiCC, bilirubin, and biliverdin—on actin dynamics have been investigated. Positively, the two phyllobilins were found to affect the organization of actin filaments in T24 and HeLa cells, whereas the heme derivatives did not show any effect. Moreover, all four compounds were able to inhibit actin polymerization and increase the depolymerization rate, with PiCC (PiCC_640(a)) generally exerting the strongest effect.

Total internal reflection fluorescence (TIRF) microscopy also demonstrated that these compounds inhibited actin nucleation, the initial step of the actin polymerization process. In silico docking studies revealed that all four compounds exhibited binding affinities to G-actin in the low micromolar range, with PiCC showing the strongest interaction. Taken together, identifying actin as a molecular target for both phyllobilins—YCC_642(a) and PiCC_640(a)—represents a key step toward understanding their bioactivity. Additionally, since T24 and HeLa are cancer cell lines, the observed anti-migratory effect may be considered a form of anticancer activity.

Another emerging area of research is the role of phyllobilins in plant defense or signaling. This family of natural products also appears to exert effects within the plant itself. Mittelberger et al. (2017) provided the first evidence that the PaO/phyllobilin pathway is relevant to pathogen-induced chlorosis in apple and apricot leaves [55]. Moreover, analyses of fungal-infected and herbivore-infested basil leaves revealed a significant increase in phyllobilin levels in the areas of pest infestation, suggesting an involvement of these chlorophyll catabolites in the plant’s defense mechanisms [56].

For example, phyllobilins are formed during the early stages of leaf yellowing (chlorosis), a process resulting from chlorophyll degradation. Importantly, early chlorosis is one of the most common symptoms of plant infection, suggesting a potential link between senescence and defense responses. This connection implies that the biochemical pathways underlying senescence-induced and pathogen-induced degreening may overlap, highlighting the dual role of phyllobilins in both developmental and stress-related processes. Therefore, investigating additional bioactivities of phyllobilins, such as antimicrobial or antiviral effects, should be a priority in order to further uncover the potential health-related impacts of these compounds.

## 5. Conclusions

Across the tested bioactive properties on phyllobilins, YCCs exhibited stronger activity than their precursors, NCCs, whereas PiCCs—the oxidation products of YCCs—appeared to display even higher activity. In light of the recently uncovered health-promoting potential of phyllobilins, these chlorophyll catabolites should no longer be regarded as irrelevant by-products of a detoxification pathway, but instead recognized as promising bioactive compounds with significant potential for future therapeutic and nutritional applications.

## 6. Future Challenges and Limitations

Since evidence suggests that PiCCs are the most bioactive compounds among the different types of phyllobilins, and given that data on these compounds remains limited, future research should focus on exploring the bioactive potential of this most oxidized phyllobilin form.

It is also important to note that the antioxidant and free radical-scavenging abilities, as well as the anti-inflammatory effects, of phytochemicals are usually the basis for other bioactivities and health benefits, including anti-diabetes, anti-obesity, blood-pressure regulation, and neuroprotective actions in neurodegenerative disorders [57]. Therefore, regarding bioactive phyllobilins, there remains a long way to go to elucidate the full spectrum of bioactivities within this family of phytochemicals and to uncover the health benefits and therapeutic effects associated with their direct dietary intake.

However, much of the work that remains to be done is largely constrained by the lack of commercially available standards, which requires researchers to undertake extensive prior identification and purification efforts. In addition, as is commonly observed in in vitro studies of biological activity, the considerable heterogeneity in experimental designs, assay conditions, and evaluation methods further complicates the direct comparison of results across different studies. This variability, which may include differences in protocols, concentrations, biological models, and analytical readouts, limits the ability to draw fully consistent or quantitative cross-study conclusions. However, this field holds clear promise for future development and is expected to yield significant scientific advances and impactful outcomes.

## Figures and Tables

**Table 1:** Naturally occurring phyllobilins identified to date.

General Name	Alternative Names
**FCC**	
**pFCC**	At-FCC *-3, Bn-FCC-2, Ca-FCC-2
**FCC_614**	At-FCC-2
**FCC_630**	At-FCC-1
**FCC_644**	Mc-FCC-62
**bc-FCC_788**	Vv-FCC-55
**hm-FCC_806**	Ma-FCC-63 and Ma-FCC-64
**hm-FCC_830**	Mc-FCC-53 and Mc-FCC-56
**hm-FCC_942**	Sw-FCC-62, Ma-FCC-69
**hm-FCC_992**	Mc-FCC-46 and Mc-FCC-49
**hm-FCC_1042**	Ma-FCC-61
**hm-FCC_1104**	hm-FCC_1105
**DFCC**	
**DFCC_618**	At-DFCC-33
**bc-DFCC_776**	Vv-DFCC-53
**NCC**	
**NCC_614**	Bn-NCC-4, At-NCC-5, Bo-NCC-2, Oe-NCC-4
**NCC_628**	So-NCC-5, Cj-NCC-2, Md-NCC-58, Pd-NCC-71, Pa-NCC-58, Oe-NCC-3
**NCC_630(a)**	Bn-NCC-3, At-NCC-2, Ej-NCC-3, So-NCC-3, Mc-NCC-49, Ps-NCC-1, Oe-NCC-1
**NCC_630(b)**	At-NCC-3
**NCC_644**	So-NCC-4, Ej-NCC-4, Sw-NCC-58, Cj-NCC-1, Pc-NCC-1, Md-NCC-49, Md-NCC-50, Mc-NCC-61, Pd-NCC-60, Ps-NCC-3, Vv-NCC-57, Pa-NCC-49, Ob-NCC-47, Ms-NCC-1, Ls-NCC, Lo-NCC, Oe-NCC-2, NCC_644
**NCC_648**	Cl-NCC-3
**NCC_662**	NCC_662
**NCC_664**	Mc-NCC-26, So-NCC-1
**NCC_678**	Pd-NCC-40, Hv-NCC-1, So-NCC-2, Ej-NCC-1, Md-NCC-35, Mc-NCC-42, Pa-NCC-35, Cl-NCC-2, NCC_678
**NCC_716**	Bn-NCC-1
**NCC_730**	Ej-NCC-2, Ob-NCC-40
**NCC_747**	Ob-NCC-35
**NCC_760**	Ob-NCC-36
**bc-NCC_788**	Ug-NCC-53, NCC_789
**NCC_792**	Bn-NCC-2, At-NCC-1, Bo-NCC-1
**NCC_806**	Pc-NCC-2, Md-NCC-47, At-NCC-4/At-mes16-NCC-1, Zm-NCC-2, Nr-NCC-2, Mc-NCC-59, Pd-NCC-56, Ps-NCC-2, Tc-NCC-2, Ug-NCC-43, Pa-NCC-47, Ms-NCC-2, Cl-NCC-4, NCC_807, NCC_806
**NCC_826**	Co-NCC-2
**hm-NCC_830**	Mc-NCC-55 and Mc-NCC-58
**NCC_840**	Pd-NCC-35, Md-NCC-31, Zm-NCC-1, Co-NCC-1, Tc-NCC1, Pa-NCC-31, Pa-NCC-33, Ug-NCC-27, Cl-NCC-1, NCC_840
**NCC_892**	Nr-NCC-1, NCC_892
**NCC_926**	NCC_926
**NCC_1002**	Pd-NCC-32, Md-NCC-29, Pa-NCC-29, NCC_1002
**DNCC**	
**DNCC_602**	At-DNCC-45 and At-DNCC-48
**DNCC_618**	At-DNCC-1/At-DNCC-33, Bo-DNCC-1
**DNCC_632(c)**	At-2HM-iso-DNCC-43
**DNCC_632(b)**	At-4HM-DNCC-41
**DNCC_632(a)**	Ej-DNCC-1, Pp-DNCC, Hvir-DNCC, Vv-DNCC-51, Pa-DNCC-45, At-mes16-DNCC-38, DNCC-632, DNCC_632
**DNCC_650**	DNCC_650
**DNCC_662**	
**DNCC_666**	Hv-DNCC, Co-DNCC-2, Ap-DNCC-1, DNCC-666, DNCC_666
**DNCC_780**	DNCC_780
**DNCC_794**	DNCC_794
**DNCC_828**	Co-DNCC-1, DNCC-828, DNCC_828
**DNCC_880**	DNCC_880
**DNCC_990**	DNCC-990
**YCC**	
**YCC_642**	Pa-YCC-54, Md-YCC-54, Cj-YCC-2, Ps-YCC-1, Pd-YCC-67, Ep-YCC-6
**YCC_676**	Ep-YCC-3
**YCC_728**	Ob-YCC-45
**YCC_762**	Ed-YCC
**bc-YCC_786**	YCC_787
**YCC_804**	Pa-YCC-51, Ps-YCC-2, Ud-YCC, Pd-YCC-61, YCC_805, Ep-YCC-5
**YCC_838**	Tc-YCC, Ep-YCC-2
**YCC_890**	Ep-YCC-4
**YCC_924**	Ep-YCC-1
**YCC_1000**	Pa-YCC-31
**YFCC**	
**hm-YFCC_1102**	YFCC_1103
**DYCC**	
**DYCC_616**	Bo-DYCC
**DYCC_630**	Vv-DYCC-63
**DYCC_678**	
**pyro-DYCC_658**	
**PiCC**	
**PiCC_640**	Md-PiCC-63
**iPB**	
**iPB_586**	Paq-iPB-55
**iPB_602**	Paq-iPB-45

* Ap: *Acer platanoides*; At: *Arabidopsis thaliana*; Bn: *Brassica napus*; Bo: *Brassica oleracea*; Ca: *Capsicum annuum*; Cj: *Cercidiphyllum japonicum*, Cl: *Citrus lemon*; Co: *Cydonia oblonga*; Ej: *Eriobotrya japonica*; Epa: *Epipremnum aureum*; Ep: *Echinacea purpurea*; Hv: *Hordeum vulgare*; Hvir: *Hamamelis virginiana*; Lo: *Liquidambar orientalis*; Ls: *Liquidambar styraciflua*; Mc: *Musa cavendish*; Md: *Malus domestica*; Nr: *Nicotiana rustica*; Ob: *Ocimum basilicum*; Oe: *Olea europaea*; Pa: *Prunus armenica*; Paq: *Pteridium aquilinum*; Pc: *Pyrus communis*; Pp: *Parrotia persica*; Ps: *Prunus salicina*; Pd: *Prunus domestica*; So: *Spinacea olearacea*; Sw: *Spathiphyllum wallissi*; Tc: *Tilia cordata*; Ud: *Urtica dioica*; Ug: *Ulmus glabra*; Vv: *Vitis vinifera*; Zm: *Zea mays*.

**Table 3:** Summary of anti-inflammatory assays performed to date on phyllobilins. Structures of the studied phyllobilins are depicted in Figure 2.

Anti-Inflammatory Capacity			
Reference	Plant Source	Tested Phyllobilins	Assay
[42]	*Urtica dioica*	YCC_804	COX-1/-2 inhibition assay
			Effect on protein expression levels of COX-2 (Western blot)
			Measurement of prostaglandin E2 concentration (ELISA assay)
[40]	*Cercidiphyllum japonicum*	YCC_642(a)	Tryptophan and kynurenine quantification
[44]	*Tropaeolum majus*	YCC_662(a), YCC_628(a)	COX-1/-2 inhibition assay
			Effect on protein expression levels of COX-2 (Western blot)

**Table 4:** Summary of anti-cancer assays performed to date on phyllobilins. The structures of the studied phyllobilins are depicted in Figure 2.

Anti-Cancer Capacity			
Reference	Plant Source	Tested Phyllobilins	Assay
[28]	NCC_644(a) from *Platanus occidentalis* and *Cercidiphyllum japonicum*; YCC_642(a) from oxidation of NCC_644(a); esters of NCC_644(a) from esterification of NCC_644(a)	NCC_644(a), YCC_642(a), esters of NCC_644(a)	Cytotoxic potential (cell proliferation assay by crystal violet staining)
		YCC_642(a)	Clonogenic assay (colony formation assay)
			Annexin V/PI staining assay
			Cell cycle analysis assay using propidium iodine staining and flow cytometry
		NCC_644(a), YCC_642(a), esters of NCC_644(a)	Cell uptake assay by cancer cells
[52]	Partial synthesis from the precursor NCC_644(a), isolated from *Cercidiphyllum japonicum*	YCC_642(a), PiCC_640(a)	Influence on cell migration (wound healing assay)

## Data Availability

The original contributions presented in this study are included in the article. Further inquiries can be directed to the corresponding authors.

## Supplementary Materials

The following supporting information can be downloaded at https://www.mdpi.com/article/10.3390/antiox15050629/s1. Table S1: Common names of the studied phyllobilins.

## Abbreviations

The following abbreviations are used in this manuscript:

COX	Cyclooxygenase
DFCC	Dioxobilin-type Fluorescent Chlorophyll Catabolite
DNCC	Dioxobilin-type Non-fluorescent Chlorophyll Catabolite
DPPH	2,2-Diphenyl-1-picrylhydrazyl
DYCC	Dioxobilin-type Yellow Chlorophyll Catabolite
FCC	Fluorescent Chlorophyll Catabolite
FRAP	Ferric reducing antioxidant power
IDO-1	Indoleamine 2,3-dioxygenase-1
iNOS	Inducible nitric oxide synthase
LPS	Lipopolysaccharide
MES16	Methylesterase family member 16
NCC	Non-fluorescent Chlorophyll Catabolite
NO	Nitric oxide
ORAC	Oxygen radical absorbance capacity
PAO	Pheophorbide *a* oxygenase
PBMC	Human peripheral mononuclear cells
pFCC	Primary Fluorescent Chlorophyll Catabolite
PiCC	Pink Chlorophyll Catabolite
RCC	Red Chlorophyll Catabolite
ROS	Reactive oxygen species
TIRF	Total internal reflection fluorescence microscopy
YCC	Yellow Chlorophyll Catabolite
