# Supplementary material for: Phyllobilins: Emerging Bioactive Chlorophyll Metabolites and Their Potential Impact on Human Health
Source: Antioxidants (Basel). 2026 May 15;15(5):629. doi: 10.3390/antiox15050629 (PMC13203533; doi:10.3390/antiox15050629)
Supplement: Supplementary file 1 [file antioxidants-15-00629-s001.zip › antioxidants-4288279-supplementary.pdf]

Table S1. Common names of the studied phyllobilins. Structures of the studied phyllobilins are depicted in Figure 2.

Phyllobilin	Common names
NCC_644(a)	So-NCC-4, Ej-NCC-4, Sw-NCC-58, Cj-NCC-1, Pc-NCC-1, Md-NCC-49 and Md-NCC-50 (epimers), Mc-NCC-61, Pd-NCC-60, Ps-NCC-3, Vv-NCC-57, Pa-NCC-49, Ob-NCC-47, Ms-NCC-1, Ls-NCC, Lo-NCC, Oe-NCC-2, NCC_644
NCC_644(a) esters (R = methyl/ethyl/butyl/octyl)	
YCC_642(a)	Pa-YCC-54, Md-YCC-54, Cj-YCC-2, Ps-YCC-1, Pd-YCC-67, Ep-YCC-6
YCC_924	Ep-YCC-1
YCC_838	Tc-YCC, Ep-YCC-2
YCC_676(a)	Ep-YCC-3
YCC_890	Ep-YCC-4
YCC_804	Pa-YCC-51, Ps-YCC-2, Ud-YCC, Pd-YCC-61, YCC_805, Ep-YCC-5
YCC_662(a)	Tm-YCC-1
pyro-YCC_618	Tm-YCC-2
YCC_646	Tm-YCC-3
YCC_628(a)	Tm-YCC-4
DYCC_616(a)	Bo-DYCC
PiCC_640(a)	Md-PiCC-63
